# 20 years experience of RhD alloimmunization at a state referral center in Rio de Janeiro: a study of 481 cases

**DOI:** 10.61622/rbgo/2025rbgo66

**Published:** 2025-10-21

**Authors:** Fernando Maia Peixoto, Ana Heloisa Nascimento Beserra, Maria Cristina Pessoa dos Santos

**Affiliations:** 1 Hospital Pedro Ernesto Rio de Janeiro RJ Brazil Hospital Pedro Ernesto, Rio de Janeiro, RJ, Brazil.; 2 Instituto Nacional de Saúde da Mulher Criança e Adolescente Fernandes Figueira Rio de Janeiro RJ Brazil Instituto Nacional de Saúde da Mulher, Criança e Adolescente Fernandes Figueira, Rio de Janeiro, RJ, Brazil.; 3 Instituto Nacional de Saúde da Mulher Criança e Adolescente Fernandes Figueira Departamento de Hemoterapia Rio de Janeiro RJ Brazil Departamento de Hemoterapia, Instituto Nacional de Saúde da Mulher, Criança e Adolescente Fernandes Figueira, Rio de Janeiro, RJ, Brazil.

**Keywords:** Anemia hemolytic autoimmune, Alloimmunization, Blood transfusion intrauterine, Pregnancy trimester first, Preganant women, Infant newborn, Infant mortality, Women's health, Health planning

## Abstract

**Objective:**

This study sought to analyze the profile of fetuses, newborns and pregnant women with alloimmunization (anti-RhD) referred to and treated over the twenty years since the launch of the Rio de Janeiro State.

**Methods:**

An observational, retrospective, descriptive study was conducted, with data obtained by analyzing the medical records of 481 mothers and their newborns. The study cohort consisted of pregnancies affected by RhD alloimmunization who delivered between March 2004 and March 2024 in Rio de Janeiro.

**Results:**

The absolute number of cases declined over the study period. Only 5.2% of the cohort began prenatal care in the first trimester of pregnancy and more than half (51.6%) of women arrived at the referral center in the third trimester. 40% of the women had prior severe HDFN. During this period, 422 intrauterine transfusions were performed on 71 fetuses, an average of 5.9 transfusions per patient. Infant mortality; was low, with 4% stillborn or evolving to neonatal mortality.

**Conclusion:**

The trend observed over the last 20 years is a reduction in the absolute number of HDFN cases which is perhaps more related to reproductive issues, particularly the sharp reduction in parity in our state, rather than to the implementation of alloimmunization prophylaxis.

## Introduction

RhD alloimmunization is an immunological condition caused by the presence of maternal anti-RhD antibodies that destroy fetal and newborn red blood cells, causing Hemolytic Disease of the Fetus and Newborn (HDFN).^(1)^ Anemia and increased serum bilirubin, due to the exacerbated hemolytic process, are responsible for the associated morbidities observed in the newborn.^(2)^ Although the fetal hyperbilirubinemia is largely resolved by placental metabolism, in severe cases the anemia and its repercussions can be fatal. Although maternal alloimmunization does not cause any clinical symptoms in the mother, HDFN has short-term and long-term complications.^(3)^

Although the incidence of HDFN has been significantly reduced due to the broad use of Rh(D) immune globulin prophylaxis, it still generates fetal mortality and morbidity including neonatal complications, and permanent neurological injuries, as well as the emotional burden for the mother and the rest of the family. Therefore the ongoing prevention of alloimmunization remains indispensable.^(4)^ There are also all the associated costs. The treatment of the disease involves neonatal hospitalization in an Intensive Care Unit (ICU), intensive use of blood products, multiple pre- and postnatal interventions, which generate an economic burden for Brazil's *Sistema Único de Saúde* (SUS), it universal healthcare system.^(5)^

The Rio de Janeiro State Program for the Prevention of Maternal Alloimmunization was established in 2003 by the Department of Health of the State of Rio de Janeiro, by Resolution No. 2154 of August 25, 2003. The goal of the program was to protect RhD negative women from alloimmunization and reduce perinatal morbidity and mortality due to Rh incompatibility.^(4)^ The program established funding and actions to coordinate the acquisition and distribution of anti-RhD immunoglobulin to SUS facilities; to provide educational materials and training about the protocol for its therapeutic use for health professionals; and to disseminate information about the risk of alloimmunization among pregnant women, all in accordance with the international guidelines that were already available in 2003.^(6)^

A study published in 2016 revealed that the persistence of alloimmunization in the state of Rio de Janeiro was multifactorial and required complex multidisciplinary measures for prenatal and gynecological care. Continuing education for health professionals and public education campaigns for the general population are strategies that can help reduce the incidence of alloimmunization.^(4)^

This study sought to analyze the profile of alloimmunized (anti-RhD) pregnant women referred to and treated at the Fernandes Figueira National Institute for Children, Women and Adolescents (IFF) and their fetuses and newborns over the twenty years since the launch of the Rio de Janeiro State Program for the Prevention of Maternal Alloimmunization.

## Methods

An observational, retrospective, descriptive study was carried out by analyzing the data abstracted from the medical records of 499 mothers and newborns who met inclusion criteria.

The study cohort consisted of 499 women whose pregnancies were affected by RhD alloimmunization, and once referred to IFF/Fiocruz received their prenatal care from the Pregnancy Care Service and delivered at the institution between March 2004 and March 2024. The Research Ethics Committee approved the project under number CAAE 70146923.3.0000.5269.

Recognizing that some conditions could introduce predictable confounding bias and, because they are relatively infrequent, would not justify subgroup analyses, the following exclusion criteria were applied: twin pregnancy, chromosomal abnormalities, malformations, and congenital infections.

**Figure 1:**
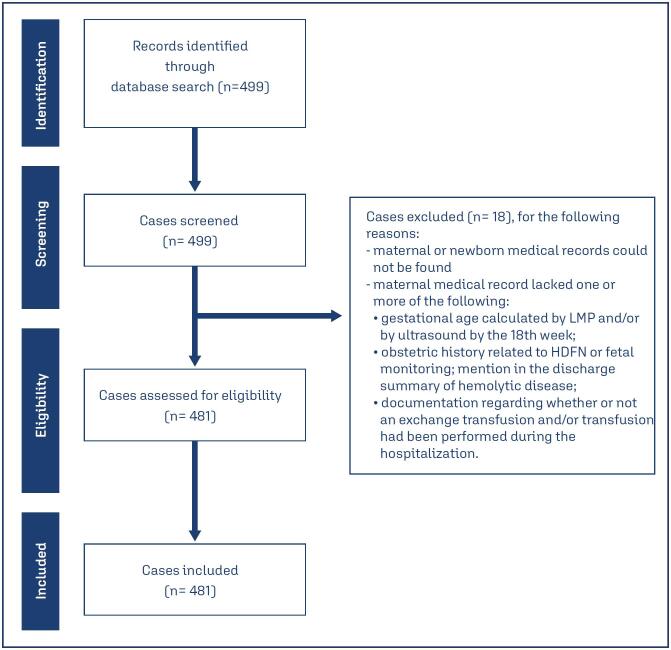
Flowchart of study selection

Eighteen (3.6%) of the 499 cases were excluded from the analysis because the maternal or newborn medical records could not be found or because the maternal medical record lacked one or more of the following: gestational age calculated by last menstrual period (LMP) and/or by ultrasound by the 18th week; obstetric history related to HDFN or fetal monitoring; mention in the discharge summary of hemolytic disease; documentation regarding whether or not an exchange transfusion and/or transfusion had been performed during the hospitalization (Figure 1).

For the 481 cases analyzed, maternal demographic characteristics, variables related to prenatal care, gestational age at the first intrauterine transfusion (IUT), total number of IUTs, gestational age at birth, days of hospitalization in the neonatal ICU, birth weight, presence of hydrops, total number of intrauterine transfusions, route of delivery, survival, death and prior history of exchange transfusion, hydrops, IUT and hospitalization in the neonatal ICU were evaluated.

## Results

**Figure 2:**
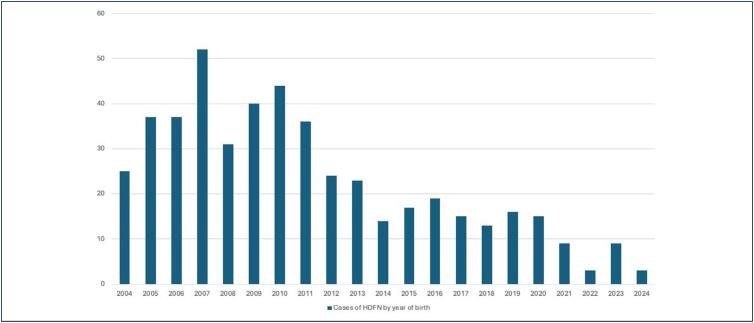
Fetuses born to pregnant women alloimmunized with anti-D antibodies in the referral centre at the Fernandes Figueira National Institute of Children, Women and Adolescents over a 20-year period from March 2004 to March 2024

The study analyzed the outcomes of 481 fetuses born to pregnant women alloimmunized with anti-D antibodies in the referral program at the Fernandes Figueira National Institute of Children, Women and Adolescents over a 20-year period from March 2004 to March 2024 (Figure 2). A clear trend toward a reduction in the absolute number of cases was observed over the period studied.

We observed that two-thirds of the alloimmunized pregnant women were multiparous, and of these, only 194 (40.3%) used immunoglobulin at the appropriate time (Table 1). Regarding when prenatal care was initiated, we found that only 25 (5.2%) started in the first trimester, and that more than half 292 (60.7%) of these women arrived at the referral center in the third trimester. Approximately 303 (63%) of the pregnant women had at least six prenatal consultations at IFF. Most women had blood type A, followed by O, B and then AB. Around 346 (72%) of pregnant women had a first Coombs value equal to or greater than 1/16. And 307 (64%) of fetuses underwent screening for fetal anemia by studying the maximum velocity of the middle cerebral artery using Doppler velocimetry.

**Table 1:** Description of characteristics related to perinatal hemolytic disease in 481 pregnancies attended between 2004 and 2024

Variable		n(%)
Maternal antecedents related to HDFN		
	Use of Immunoglobulin	194(40.33)
	Previous Stillbirth	85(17.67)
	Previous Neonatal Mortality	68(14.13)
	Previous Hydrops	9(1.87)
	Previous Intrauterine Transfusion	33(6.86)
Arrival at reference center		
	First Trimester	25(5.19)
	Second Trimester	164(34.09)
	Third Trimester	292(60.7)
Fetal		
	Intrauterine Transfusion (IUT)	71(14.76)
	Number of IUT per Case	27(5.7)
	Hydrops	16(3.32)
	Stillbirth	9(1.87)
Newborn		
	Prematurity (<37 weeks)	230(47.81)
	Hydrops	15(3.11)
	Exchange Transfusion	75(15.59)
	Neonatal Death	10(2.07)
	Variable	Value
	Total	481(100)

We found that 194 (40.3%) of pregnant women had already experienced a history of perinatal hemolytic disease with cases of hydrops, stillbirths, and neonatal deaths. In addition, approximately 33 (6.8%) had already undergone intrauterine transfusion in a previous pregnancy. During this 20 years period, 422 intrauterine transfusions were performed on 71 fetuses, with an average of 5.9 procedures per patient (range: 1 to 14). Of these transfusions, 398 (82.7%) were performed by the same professional (FMPF). Nearly half 280 (48%) of newborns were born prematurely. And 293 (61%) of deliveries whereby Cesarean section.

Regarding the index pregnancy of the women studied, we observed that 19 (3.94%) evolved to stillbirth or neonatal mortality. We also observed that 134 (28%) required cardiopulmonary resuscitation maneuvers. Hydrops was observed in only 16 (3.3%) of cases. After birth 75 (15.5%) required exchange transfusions, of which 62 (13%) required only one exchange transfusion. Regarding the length-of-stay in the neonatal ICU, one-third about 159 (33%) newborns were in the unit for up to 5 days.

## Discussion

HDFN is characterized by a decrease in the half-life of red blood cells in the fetus or newborn due to the action of maternal antibodies due to alloimmunization.^(2)^

Alloimmunization is the production of antibodies from antigens that are not specific to that individual, and we know that several antigens can cause HDFN, such as those from the ABO, Rh, Kell, Duffy, MNS, Kidd, Diego, Colton and other systems.^(7)^ In the Rh system, the D antigen is the most immunogenic and is usually responsible for 90% of cases of hemolytic disease in our setting.^(5)^

Maternal alloimmunization by the Rhesus D (RhD) antibody is a preventable process, through the administration of anti-RhD immunoglobulin in pregnant women who are RhD negative, if administered in the appropriate dose and at the appropriate time.^(8-10)^ This study sought to analyze the profile of alloimmunized pregnant women (anti-RhD) referred to and treated at the Fernandes Figueira National Institute for Children, Women and Adolescents (IFF) as well as their perinatal outcome during the first twenty years of a state health department program established in 2003 for the prevention of maternal alloimmunization.

The results reflect issues related to the organization of the perinatal network in the State of Rio de Janeiro. The delay in the arrival of previously alloimmunized patients to the referral center for high-risk prenatal care was clearly demonstrated, with only 5.6% starting prenatal care in the first trimester, and with more than half arriving in the third trimester. It is important to emphasize that more than half of the patients had two or more previous pregnancies; multiparity is clearly related to the greater potential severity of the disease.^(11)^ Another recurring failure in the care of these patients is the issue of Rh(D) prophylaxis in a timely and appropriate manner.^(10,12,13)^ Our findings demonstrate that less than half received immunoglobulin at the appropriate time, which also reflects an important gap to be filled in the continuing education of health professionals and the pregnant women themselves regarding the need for and timing of prophylaxis.^(14)^

The study also found that most newborns had good outcomes, but required intensive use of technology, such as specialized fetal medicine care, intrauterine transfusions, and admission to the neonatal ICU. Those who required admission to the neonatal ICU remained there for an average of 7 days. We also found that the number of cases involving hydrops, exchange transfusions, and intrauterine transfusions decreased over the years, but this does not mean that alloimmunization and hemolytic disease had decreased up to 2011 based on other research at our center.^(15,16)^

The advances seem to be more related to improvements in the monitoring and treatment of severe cases at the reference center and less to the prophylaxis of alloimmunization. Based on our data, women continue to become sensitized, arriving late for specialized care and generating perinatal repercussions despite the advances of the Rio de Janeiro State Department of Health's Program for the Prevention of Maternal Alloimmunization established in 2003. In this context, future studies should consider the role of the reduction in fertility already demonstrated in Brazil,^(17)^ partly a consequence of the Zika virus "scare" in 2016 and the Coronavirus (Covid-19) pandemic 2020-2022. The resulting dramatic decline in the birth rate in the State of Rio de Janeiro likely has contributed to a declining number of affected newborns – and less severe cases – over the past 20 years.

During the study period, there were no changes in the referral and counter-referral system for alloimmunization cases in the state of Rio de Janeiro, nor was there the opening of a public fetal medicine center that could account for the magnitude of the reduction in cases managed at IFF.

Some limitations and strengths of this study should be considered. First, it was not possible to follow up these women and babies for long-term. However, we analysed data until birth and puerperium. Second, our study involved pregnancies affected by RhD alloimmunization only in Rio de Janeiro state and this could not reflect the whole country, Brazil. Due to these cases included we have considered that we could have some difference between state within the country. Finally, the observed the decrease of parity in Brazil and consequently this impact in the small number of pregnancies affected by RhD alloimmunization. Nonetheless, we have to highlight that is the first study developed in South America and hence Brazil to analysed this data over the years.

## Conclusion

Analyzing the profile of alloimmunized pregnancies (anti-RhD) and their repercussions at our referral center – taking as the time frame the twenty years since the implementation of the Rio de Janeiro State Health of Department's Program for the Prevention of Maternal Alloimmunization in 2003 – we observed that although advances in highly specialized care have resulted in lower perinatal morbidity and mortality, women continue to become sensitized and continue to arrive late for specialized care, generating what are likely avoidable perinatal repercussions. The trend observed over the last 20 years is a reduction in the absolute number of HDFN cases which is perhaps more related to reproductive issues, particularly the drastic reduction in parity in our state, rather than related to the implementations of alloimmunization prophylaxis.

## Data availability

: The authors did not make the data from this article available in repositories prior to submission.
